# CSpace: a concept embedding space for biomedical applications

**DOI:** 10.1093/bioinformatics/btaf376

**Published:** 2025-06-27

**Authors:** Danilo Tomasoni, Luca Marchetti

**Affiliations:** Fondazione The Microsoft Research—University of Trento Centre for Computational and Systems Biology (COSBI), 38068 Rovereto (TN), Italy; Fondazione The Microsoft Research—University of Trento Centre for Computational and Systems Biology (COSBI), 38068 Rovereto (TN), Italy; Department of Cellular, Computational and Integrative Biology (CIBIO), University of Trento, 38123 Povo (TN), Italy

## Abstract

**Motivation:**

The rise of transformer-based architectures has dramatically improved our ability to analyze natural language. However, the power and flexibility of these general-purpose models come at the cost of highly complex model architectures with billions of parameters that are not always needed.

**Results:**

In this work, we present CSpace: a concise word embedding of biomedical concepts that outperforms all alternatives in terms of out-of-vocabulary ratio and semantic textual similarity task, and has comparable performance with respect to transformer-based alternatives in the sentence similarity task. This ability can serve as the foundation for semantic search by enabling efficient retrieval of conceptually related terms. Additionally, CSpace incorporates ontological identifiers (MeSH, NCBI gene and taxonomy IDs), enabling computationally efficient disease, gene or condition relatedness measurement, potentially unlocking previously unknown disease-condition associations.

**Availability and implementation:**

Full and compressed models are available on Zenodo at https://doi.org/10.5281/zenodo.14781672, while training code, examples, interactive visualizations and experiments are available at https://doi.org/10.5281/zenodo.15125706 and on the GitHub repository.

## 1 Introduction

The rise of deep learning methods allowed for types of text analysis that were not possible before. Word embeddings built with deep learning methods are based on the Distributional Hypothesis (Harris 1954), which states that words that appear in the same contexts tend to have similar meanings. Good embeddings are essential both for single-word and composite words (n-grams) semantic similarity tasks as well as a building block of many natural language processing (NLP) tasks such as relation extraction and semantic text similarity.

Previous attempts have been made to improve the quality of word embeddings for biomedical text, by selecting the best hyperparameters (Chiu *et al.* 2016, Dupré *et al.* 2018), including MeSH ontology terms in the training process (Zhang *et al.* 2019) and by exploiting mesh concepts co-occurrence for fine-tuning the resulting word embeddings (Noh and Kavuluru 2021). These attempts, however, model only a relatively small number of single words, while concepts are often expressed as a complex set of multi-word expressions.

Further, even if it is known that training algorithm hyperparameters affect the embedding quality (Dupré *et al.* 2018), to the best of our knowledge, they are rarely optimized directly.

Recently, an alternative approach based on the transformer architecture (Vaswani *et al.* 2017) emerged to embed sentences and paragraphs directly (Neelakantan *et al.* 2022), (Sturua *et al.* 2024), but such alternatives are not well suited to embed single concepts and require specialized hardware to be trained and even run.

The model herein presented has been developed taking into consideration these concerns. We built a concept embedding model by (i) preprocessing the input text before the actual training to reduce synonym variability, (ii) optimizing hyperparameters on the training corpus to maximize performances on manually curated test sets (Pakhomov *et al.* 2010), (Pedersen *et al.* 2007), and (iii) performing training data augmentation by deriving composite-word concepts with Pubtator3 (Wei *et al.* 2019, 2024) and statistical co-occurrence of words (Mikolov *et al.* 2013b).

Selected hyperparameters maximize the correlation between model and human similarity judgements on three manually curated test sets: the Human-level Similarity Reference Standard (UMNSRS) for similarity and relatedness (Pakhomov *et al.* 2010), which is composed of two datasets of relatedness and similarity scores manually curated by the domain experts from the University of Minnesota Medical School, and the MayoSRS (Pedersen *et al.* 2007), a set of 101 medical concept pairs manually rated by medical coders for semantic relatedness.

Further, we derive an embedding representation also for chemical and disease MeSH IDs, species taxonomy IDs, and NCBI gene IDs in a completely unsupervised setting by replacing annotated text with the related ontological ID in the sentences.

We argue that the combination of text preprocessing, text augmentation, and hyperparameters optimization enables CSpace to outperform all other embedding models in both concept and sentence similarity tasks. Notably, it surpasses the popular transformer-based OpenAI ada-v2 model in the concept similarity task, with a performance trade-off of less than 5% in the sentence similarity task. This is particularly noteworthy given that CSpace operates and can even be trained without the need for specialized hardware such as GPUs or TPUs. Additionally, it utilizes less than 10% of the embedding dimensions required by ada-v2, making it a highly efficient and accessible tool for democratizing advanced embedding technologies beyond large corporate environments.

## 2 Materials and methods

The source text used to train the embeddings included the full PubMed titles and abstracts (>35 Mln), PubMedCentral titles, abstracts, bodies, and figure/table captions (>337.000), ClinicalTrials.gov brief titles, brief summaries and detailed descriptions (>428.000), the preprint titles, abstracts, bodies, and figure/table captions published in BioRxiv (>20.000) and MedRxiv (>20.000) published in 2024. To provide a comprehensive performance analysis of our tool with training datasets of increasing size, we present results based on two versions of the literature corpus, updated as of February 2023 and August 2024, respectively.

For each corpus and for each article, each paragraph has been preprocessed by splitting it into sentences, removing punctuation symbols, parentheses, and special characters, and normalizing remaining words through lemmatization and lowercase. This has been done with the popular SpaCy natural language processing toolkit (Honnibal *et al.* 2017) and resulted in a plain-text file with a list of normalized sentences, that will be used as a base for the training dataset. We will refer to the output of this pre-processing step as the mono-gram dataset featuring 1.5 billion sentences with an average of 21 words per sentence.

To such a base dataset, we added sentence variants where (i) highly co-occurring words and (ii) words annotated in Pubtator3 (updated up to August 2024) as the same entity are joined together by a conventional “_” sign. This allows us to learn word embeddings for biomedical concepts that are often expressed as a complex set of multiple words.

Finally, we computed a unique identifier version for each sentence by replacing the Pubtator3-annotated text with the following syntax:

<entity-type>_<entity-id>

where entity-type is the type of annotation (gene, disease, chemical, mutation, species) and entity-id is the ontology identifier (e.g. 9096 is “Human,” mesh_d005356 is “Fibromyalgia”).

Thus, the resulting ontology concepts will have, for example, the following form: species_9096 for Human, disease_mesh_d005356 for Fibromyalgia.

We used the popular GenSim Phrases submodule (Řeh ůřek and Sojka 2010, Mikolov *et al.* 2013b) to automatically detect co-occurring words (bi-grams) and Pubtator3 as a source of annotated text to detect multiple words that form a single concept (n-grams) and their corresponding ontology identifiers. Every paragraph was augmented with all these variants to obtain our n-gram annotated dataset, containing 3.2 billion sentences with an average of 21 words per sentence and 6.4 Mln multi-word concepts, 500 000 ontology identifiers (subdivided into 216 000 genes, 181 000 species, 12 000 diseases, and 91 000 chemical elements). This means that the proposed data augmentation approach expands the mono-gram dataset by roughly a factor of 2.1. After randomly shuffling the sentences, the resulting dataset is used for training CSpace.

This data augmentation procedure allows us not only to improve CSpace performances but also to reduce the out-of-vocabulary ratio (OOV) (Table 1) for single, multi-word, and ontology concepts, which is critical for the real-world applicability of the model outside the gold-standard corpora in the task of synonyms suggestion, concept, and sentence similarity.

**Table 1.** Out of vocabulary ratio on different benchmark datasets.

Dataset	CSpace	BioWord2Vec	BERT-CRel-all
MayoSRS	**12.87** (13.86)	64.35	67.32
UMNSRS rel	**3.74** (3.74)	9.36	12.94
UMNSRS sim	**2.30** (2.30)	7.95	11.30

CSpace was evaluated with training data up to February 2023 (shown in parentheses) and August 2024. Regardless of the version, it has the lowest percentage of out-of-vocabulary (OOV) words across all datasets compared with publicly available alternatives (values highlighted in bold).

CSpace was trained with the FastText algorithm (Bojanowski *et al.* 2016), an improvement over Word2Vec (Mikolov *et al.* 2013a) that exploits sub-word similarities among different concepts to improve the embedding quality (ex. “deltaproteobacteria” and “proteobacteria” share a large sub-word and thus in FastText have similar embeddings).

Word2Vec is a self-supervised learning strategy to derive an N-dimensional vector representation from natural language words. It works by training an artificial neural network (ANN) with one hidden layer on the task of predicting a configurable number of words surrounding the word of interest (this training objective is named Skip-gram). The underlying idea is that to complete this task, the ANN should store in the hidden layer a numerical representation resembling the “meaning” of the word of interest. At the end of the training, the ANN is discarded, and only the hidden layer is retained: it is the N-dimensional representation of the meaning of the original word, namely its embedding.

**Figure 1.**
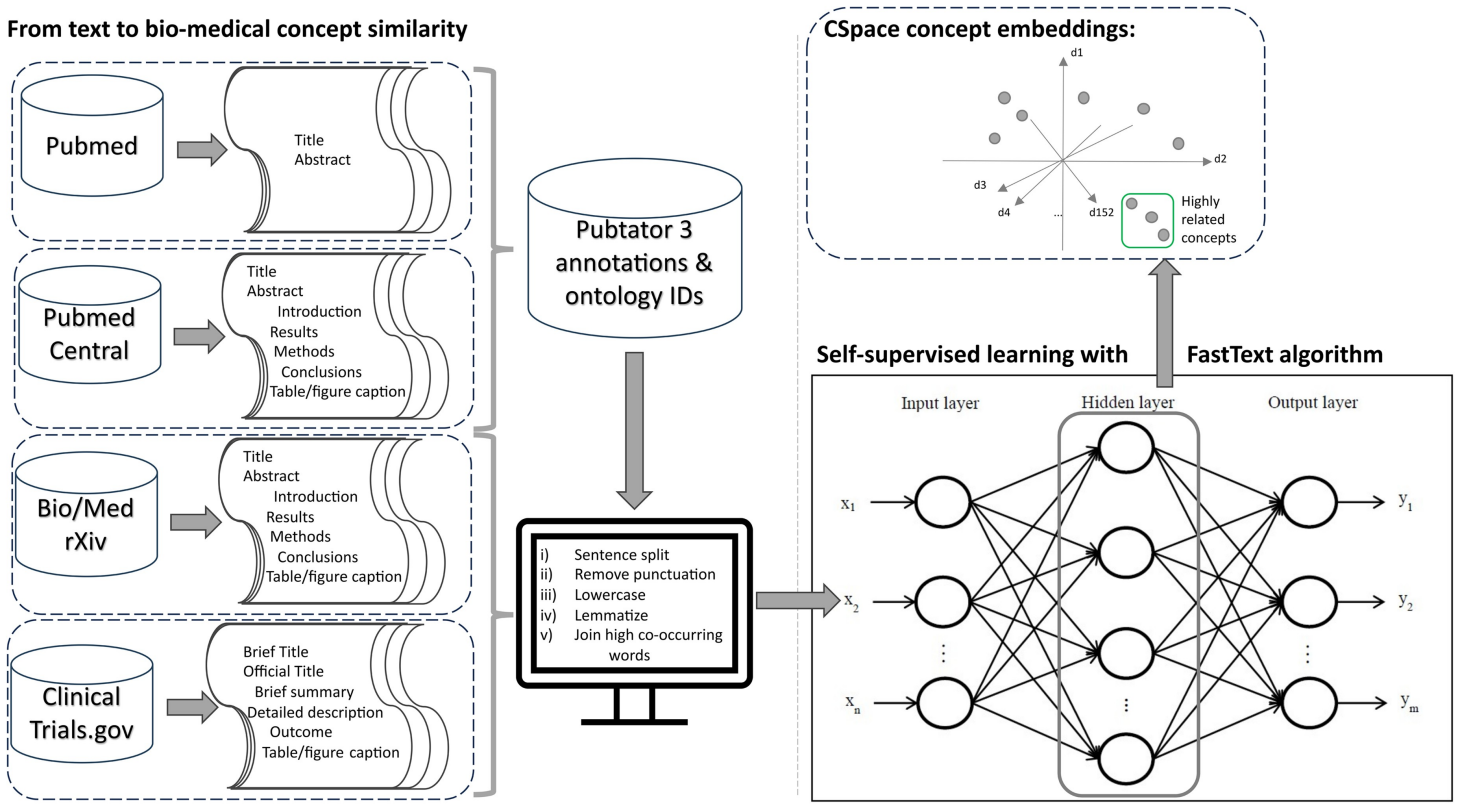
A complete overview of the methods and the data employed to build CSpace.

In Table 2, we report the hyperparameters used to train CSpace, while in the Supplementary Methods (Supplementary File S1, available as supplementary data at *Bioinformatics* online), we discuss their meaning, the final chosen value, and explain the validation metrics. A complete overview of the CSpace training process is depicted in Fig. 1, and the code implementing the data pre-processing and model training is freely available on GitHub, see the Data Availability section.

**Figure 2.**
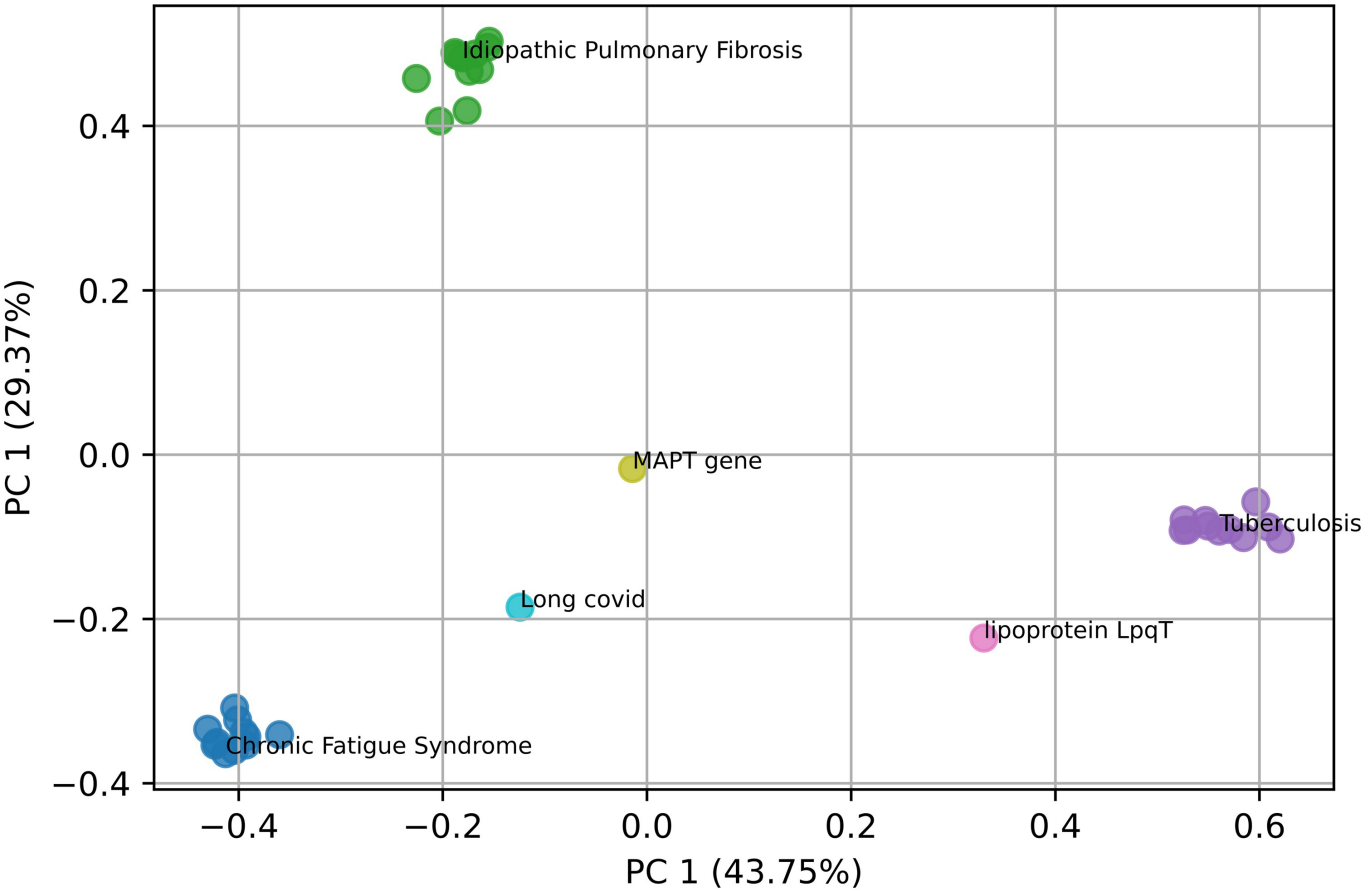
PCA of embeddings for CFS, IPF, and tuberculosis. The top 10 most similar concepts are depicted with the same color. MAPT gene (causally associated with both Long Covid and IPF), Long Covid (sharing symptoms with CFS), and lipoprotein LpqT (enhances survival of mycobacterial Tuberculosis *in vivo* or *in vitro*) are closer in embedding space to the related disease.

**Table 2.** Hyperparameters used to train CSpace (FastText hyperparameter) and the word co-occurrence model (phrases hyperparameter).

FastText hyperparameter	Value
α	0.03
min α	0.0001
Negative examples	5
Skip-Gram	True
Window size	20
Vector size	152
Max length of char n-grams	6
Min length of char n-grams	3
Min word frequency	5
Exponent for the negative sampling distribution	0.75
Down-sample threshold	1e-5
Number of epochs	5
Number of parallel workers	50
Phrases hyperparameter	Value
Threshold (for training data augmentation)	1
Threshold (for test)	5

All parameters not explicitly mentioned are set to the default value (package version: gensim 4.1.7).

## 3 Results

We validated CSpace with special care to minimize out-of-vocabulary (OOV) words: a richer vocabulary is important to accurately model biomedical sentences from different contexts. In Table 1, we showed that the CSpace OOV word ratio is much lower with respect to other models, thanks mainly to our corpus size and the data augmentation technique that also exploits Pubtator3 named entities.

This is an important point to consider when comparing the performances because the test procedure excludes OOV words from the correlation computation, thus potentially biasing the results towards models with a low number of high-quality concept vectors.

To showcase CSpace embedding properties, we considered as case studies the diseases “Chronic Fatigue Syndrome” (CFS), “Idiopathic Pulmonary Fibriosis” (IPF) and “Tuberculosis.”

In Table 3, we show the correlation matrix between both semantically equal and slightly different concepts in the context of CFS. We notice that almost-equivalent concepts, such as “chronic fatigue,” “fatigue syndrome,” and “chronic fatigue syndrome,” have a cosine similarity near to 1 (>0.9), a highly desirable property. CSpace also has a high cosine similarity (>0.8) with strictly related concepts such as “Myalgic Encephalomyelitis” (ME), that have considerable symptoms overlap with CFS, often referred to in the literature as “Myalgic Encephalomyelitis/Chronic fatigue syndrome” (ME/CFS) allowing for a computationally cheap and effective disease-condition relatedness measurement. CSpace also retains a high cosine similarity (≥0.7) with ontology identifiers that are closely related to the concept, such as MeSH: D015673 (encoded in CSpace as disease_mesh_d015673).

**Table 3.** Example correlation matrix.

	CFS	CF	FS	ME	D015673
CFS	1				
CF	0.95 (0.93)	1			
FS	0.98 (0.97)	0.93 (0.93)	1		
ME	0.87 (0.81)	0.82 (0.74)	0.85 (0.79)	1	
D015673	0.80 (0.72)	0.78 (0.67)	0.77 (0.68)	0.71 (0.61)	1

Example of cosine similarity between strictly related and equivalent concepts “Chronic Fatigue Syndrome” (CFS), “Chronic Fatigue” (CF), “Fatigue Syndrome” (FS), “Myalgic Encephalomyelitis” (ME) and CFS MeSH ID, from CSpace with training data up to August 2024 and February 2023 (shown between parentheses).

To further evaluate the quality of the embeddings, Principal Component Analysis (PCA) was applied to the local neighborhoods of CFS, IPF, and Tuberculosis within CSpace. The resulting projections reveal distinct clusters, with semantically related concepts—such as MAPT, Long Covid, and lipoprotein LqpT—positioned near their associated diseases (see Fig. 2). Notably, MAPT appears midway between IPF and CFS, even though it is not directly linked to CFS in the literature. This positioning results from MAPT’s strong association with Long Covid, which, in turn, is linked to CFS. These findings highlight the semantic coherence exhibited by the CSpace embeddings.

An example of CSpace ability to retrieve semantically related concepts is shown in Table 4, where diseases with a significant symptom overlap with “Fatigue Syndrome, Chronic” (MeSH: D015673) are correctly extracted from the embedding space. Note that there is considerable symptom overlap between “Chronic Fatigue Syndrome” and “Fibromyalgia,” and indeed, “Fibromyalgia” appears multiple times in the list, alone or together with other highly related ontology IDs.

**Table 4.** Most similar concepts to MeSH “Fatigue Syndrome, Chronic.”

MeSH ID	MeSH term	CSpace similarity
D015673+D005356	Fatigue Syndrome + Fibromyalgia	0.900
D005356	Fibromyalgia	0.890
D000092202	Exercise-Induced Allergies	0.866
D018923	Persian Gulf Syndrome	0.856
D051271	Headache Disorders, Secondary	0.851
D011602	Psychophysiologic Disorders	0.846
D005221	Fatigue	0.846
D015535+D005356	Arthritis, Psoriatic + Fibromyalgia	0.844
D043183+D005356	Irritable Bowel Syndrome + Fibromyalgia	0.841
D056770+D017116	Netherton Syndrome + Low Back Pain	0.837
D002058	Burns, Electric	0.836
D015212+D005221	Inflammatory Bowel Diseases + Fatigue	0.835
D012019	Reflex Sympathetic Dystrophy	0.834
D059352	Musculoskeletal Pain	0.833
D005222	Mental Fatigue	0.832
D002057	Burns, Chemical	0.829
D018771	Arthralgia	0.828
D054972	Postural Orthostatic Tachycardia Syndrome	0.828
D006261	Headache	0.828
D018781+D001007	Tension-Type Headache + Anxiety	0.828
D002054	Burning Mouth Syndrome	0.827
D018777	Multiple Chemical Sensitivity	0.825

Most similar concepts to “Fatigue Syndrome, Chronic” (MeSH: D015673) in vector space with CSpace, up to cosine similarity 0.825. All diseases reported share some clinical phenotype of Fatigue Syndrome, Chronic. CSpace was trained with data up to August 2024.

We performed similar experiments on the diseases “Idiopathic Pulmunary Fibrosis” (Supplementary File S2, available as supplementary data at *Bioinformatics* online, Supplementary Tables S1 and S2, available as supplementary data at *Bioinformatics* online) and “Tuberculosis” (Supplementary File S2, available as supplementary data at *Bioinformatics* online, Supplementary Tables S3 and S4, available as supplementary data at *Bioinformatics* online). The results were comparable to those reported for “Chronic Fatigue Syndrome” providing further evidence that the findings presented here are not biased by focusing on a specific disease. Most notably, the NCBI taxonomy ID for “Mycobacterium Tuberculosis,” the bacteria responsible for the Tuberculosis, was retrieved as its second most related ontological concept showing that CSpace can be used effectively to compare concepts from different ontologies: a valuable property that can be used, for instance, to retrieve genes most related to a disease of interest, (see Supplementary File S2, available as supplementary data at *Bioinformatics* online, Supplementary Table S5, available as supplementary data at *Bioinformatics* online, for some examples).

CSpace performances were also rigorously assessed on the concept and sentence similarity tasks with standard benchmarks.

With respect to the concept similarity, we tested CSpace against previously published models and OpenAI ada-v2 on three widely-used benchmarking datasets: (i) UMNSRS for similarity (Pakhomov *et al.* 2010), (ii) UMNSRS for relatedness (Pakhomov *et al.* 2010), and (iii) MayoSRS (Pedersen *et al.* 2007).

The testing procedure includes three steps: (i) compute the cosine similarity score for every combination of term pairs and ontology concept pairs, where ontology concepts are identified by their UMLS Concept Unique Identifiers (CUIs), (ii) measure the Pearson’s correlation coefficient between the computed scores and those provided by human experts, and (iii) compare results with other state-of-the-art embeddings (Zhang *et al.* 2019), (Noh and Kavuluru 2021).

The comparison results are summarized in Table 5: we note that CSpace provides ontology identifiers embeddings not available in any other model. Furthermore, CSpace reaches superior performances with respect to the other word embedding models BERT-CRel-all (Noh and Kavuluru 2021) and BioWord2Vec (Zhang *et al.* 2019), despite the fact that they contain, respectively, around 0.333 Mln and 2.32 Mln single-word vectors, while CSpace contains ∼30 Mln concept vectors.

**Table 5.** Word and concept similarity model comparison.

Dataset	OpenAI ada-v2	BERT-Crel-all	BioWord2Vec	CSpace
Mayo	0.33	0.65	0.6	**0.69 (0.69)**
UMN rel	0.42	0.64	0.62	**0.66** (0.63)
UMN sim	0.45	0.68	0.67	**0.71** (0.68)
Mayo (ID)	NA	NA	NA	**0.81** (0.79)
UMN rel (ID)	NA	NA	NA	0.51 (**0.52**)
UMN sim (ID)	NA	NA	NA	0.60 (**0.61**)

We measured the Pearson correlation coefficient between human and model similarity judgement (expressed as embedding vectors cosine similarity) on UMNSRS and MayoSRS datasets. Highlighted in bold is the best correlation among all the models. Ontology MeSH IDs are available only in CSpace updated with training data up to February 2023 (between parentheses) and August 2024. The high correlation in “Mayo (ID),” a test set composed of many multi-word expressions, suggests an advantage in modelling concepts rather than words.

With respect to the sentence similarity, CSpace can effectively encode sentences through an algorithm that consists of five main parts: (i) sentence preprocessing, with the same procedure carried out during the training phase, (ii) join n-grams with “_” sign through a word co-occurrence model (Mikolov *et al.* 2013b) and, optionally, entities annotation (Wei *et al.* 2024), (iii) convert n-grams to CSpace embeddings, and (v) apply the Word Mover Distance (WMD) algorithm. In Table 6, we show sample sentences with their distance score obtained with the after mentioned procedure.

**Table 6.** Examples of sentence similarity with CSpace.

Sentence 1	Sentence 2	WMD
Table 5 shows the distribution of patients with covid-19.	Table 1 shows the baseline characteristics of COVID-19 patients.	0.241
Studies into long COVID suggest many overlaps with ME/CFS	Twenty-five out of 29 known ME/CFS symptoms were reported by at least one selected long COVID study.	0.368
A benchmark data set of sentence pairs from the biomedical literature is manually annotated by five human experts.	In order to provide a reliable test-bed for this experiment, we generate a dataset of 1000 pairs of sentences from biomedical publications that are annotated by ten human experts.	0.452
Mean serum or sera concentration time profiles of mRNA-encoded protein product.	A dose-dependent concentration of human IgG in mouse serum was observed, as expected (Fig. 1).	0.603
The results are the mean of serum antibody concentration.	All data are presented as relative mean values ± standard deviation against background signals from plates without the addition of sera.	0.649
Heart failure (HF) is a complex clinical syndrome resulting from diverse primary and secondary causes and shared pathways of disease progression, correlating with substantial mortality, morbidity, and cost.	M-wave alterations were greater in ME/CFS patients as in those with long-COVID when the highest muscle strength and highest exercise performance were measured.	0.779

Plain text is preprocessed to remove symbols and stop-words, and n-grams are joined with the “_” sign. The resulting *n*-grams are converted to CSpace embeddings that are then used to compute the WMD. See (Kusner *et al.* 2015), (Flamary *et al.* 2021), the “Data Records” and “Usage Notes” sections for a precise description of the algorithm.

To formally evaluate CSpace performance on sentence similarity, we employed the BIOSSES dataset (Soğancıoğlu *et al.* 2017), consisting of 100 sentence pairs from the biomedical literature, manually annotated by five human experts with a rank score from 0 (completely dissimilar) to 4 (semantically equivalent). The average of the rank scores is then used as a target similarity measure. To compute sentence similarity starting from concept embeddings, we used three methods, whose results are summarized in Table 7: (i) direct generation of sentence embedding, (ii) weighted average of sentence word embeddings following (Sanjeev *et al.* 2017), and (iii) the WMD algorithm (Kusner *et al.* 2015), (Flamary *et al.* 2021) applied on concept embeddings.

**Table 7.** Pearson correlation on BIOSSES dataset.

Model	Direct	Weighted average	WMD
OpenAI ada-v2	**0.804**	NA	NA
CSpace	NA	0.742 (**0.816**)	0.769 (**0.785**)
BioWord2Vec (intrinsic)	NA	0.740	0.759
BioWord2Vec (extrinsic)	NA	0.731	0.743
BERT-CRel-all	NA	0.621	0.704

Correlation between human similarity judgement and model similarity judgement following different algorithms for sentence embedding generation. The direct generation of sentence embedding is supported only by ada-v2. Weighted average of word embeddings following (Sanjeev *et al.* 2017), and WMD was applied to the other models. The general purpose ada-v2 has almost the same performance of CSpace in the BIOSSES dataset despite being much more complex. CSpace shows superior performance with respect to other word similarity models using the WMD algorithm (values highlighted in bold). CSpace was trained with data up to February 2023 (between parentheses) and August 2024.

Note that in the weighted average (Sanjeev *et al.* 2017), the normalizing constant “A” was set to 3e-4. In our experiments this value had consistently better correlation performances on all the tested models with respect to the value reported in the paper (1e-4). Note also that the correlation between the human judgement and WMD distance is negative because the former is a similarity measure, and the latter is a distance measure. To transform the distance measure into a similarity measure, we multiplied the distance measure by −1 before computing the correlation. The results in Table 7 show that CSpace has a higher correlation with human similarity judgement with respect to other word embedding models when applying the WMD algorithm.

Further, we compared CSpace with the popular, transformer-based, GPU-intensive, and cloud-hosted ada-v2 (Neelakantan *et al.* 2022) OpenAI embeddings model. According to the results in Table 7, the performance penalty is lower than 5%. This is particularly noteworthy considering that CSpace can be run and even trained without specialized GPUs or TPUs, has less than 10% of the ada-v2 embedding dimension (vector size of CSpace is 152, compared to 1536 of ada-v2), and was generated with a simple WMD algorithm, while ada-v2 is a complex deep learning model with many hidden parameters to be learned. The consistently high correlations with human judgement, regardless of the relatively low number of dimensions employed compared to other alternatives, suggest that performance does not necessarily scale with the number of embedding dimensions. A similar result was also observed by Jina embedding authors in (Sturua *et al.* 2024).

The provided examples, together with the benchmark results, suggest that CSpace can be used to (i) generate semantic synonyms for a given concept, (ii) explore similar diseases with multiple common clinical manifestations, (iii) explore genes most associated with a given disease, (iv) quantify the semantic relatedness of different biomedical sentences, and (v) associate free-text concepts to semantically similar ontological identifiers, a process commonly known as “entity linking” within the context of knowledge graph construction.

We conducted similar experiments on a compressed version of CSpace (See Data Availability) that is about 99.9% smaller than the original model and retains only the top 100K high-frequency concepts. With the compressed model, there is a performance gain on the concept similarity task (2% on Mayo SRS, 1% on UMNSRS relatedness, and 1% on UMNSRS similarity), while the performance penalty on the sentence similarity task, measured on the BIOSSES dataset, is about 11%.

## 4 Discussion

CSpace word embeddings were trained mainly for intrinsic tasks. In intrinsic tasks, embeddings are directly used for the end prediction, while in extrinsic tasks, embeddings are included in a larger deep-learning pipeline.

According to (Zhang *et al.* 2019), if the hyperparameter “window size” is high (>15), the resulting embeddings will be more suited to intrinsic tasks, while if the window size is relatively small (∼5), it is more suitable for extrinsic tasks.

However, the recent trend in Sentence Text Similarity tasks is to employ transformer-based architectures to learn contextual embeddings (rather than context-free, unique concept embedding) directly as a part of the deep-learning pipeline without building upon a pre-trained embedding model (Vaswani *et al.* 2017, Devlin *et al.* 2019). For this reason, we build an embedding model for intrinsic tasks only, showing that, nonetheless, it can be used for sentence text similarity.

Note also that CSpace embeds concepts and ontology IDs rather than words, while every other alternative that we are aware of embeds single words only. This opens the possibility to effectively and efficiently measure not only the similarity between biomedical concepts and sentences, but also associations among diseases, genes, and clinical conditions, even across different ontologies. For instance, the NCBI taxonomy ID for “Mycobacterium Tuberculosis,” the bacterium responsible for tuberculosis, was retrieved as the second most related ontological concept to the MESH ID for “Tuberculosis”. Similarly, “Long Covid” exhibited a high cosine similarity (>0.8) with “Myalgic Encephalomyelitis,” reflecting their shared symptoms. Finding such high-similarity concepts is extremely efficient even if CSpace contains ∼30 million vectors because this operation under the hood is performed as a single matrix multiplication exploiting modern CPU data-parallelism. If memory usage is a concern, the model can be compressed to achieve the desired memory/performance tradeoff.

An additional application of CSpace embeddings is the retrieval of genes most closely associated with a given disease of interest. We used CSpace to learn about genes closely associated with “Idiopathic Pulmonary Fibrosis” (IPF) by filtering the closest embeddings by gene identifier. The top-resulting gene was MAPT, and a subsequent literature search showed that it is causally associated with IPF (Zhang *et al.* 2024). We summarized this and other similar findings in Supplementary File S2, available as supplementary data at *Bioinformatics* online, Supplementary Table S5, available as supplementary data at *Bioinformatics* online. Another potential application is semantic entity linking. When constructing knowledge graphs, a common challenge is linking concepts expressed in free text to a standardized ontology. The joint embedding of free-text concepts and ontology identifiers in CSpace enables the retrieval of the ontology concept most closely related to the given free-text input, without relying on approximate string overlap heuristics (Neumann *et al.* 2019).

We validated CSpace on the synonym suggestion, concept similarity, and sentence similarity tasks. Synonym suggestion is especially important in information-retrieval applications, where retrieving all the different ways a concept can be expressed helps ensure that no relevant results are missed. Concept similarity aims at determining a similarity score between two concepts, the higher the score, the more the concepts are related. This is important to evaluate the quality of the word embeddings, that is, verifying the correspondence between human similarity judgement and high cosine similarity in the embedding space. Sentence similarity is also critical in biomedical and clinical domains to organize and cluster together relations derived from scientific literature, a base building block needed to derive knowledge from a large unstructured body of publications.

CSpace aims to be parsimonious in the embedding dimensions: BERT-CRel-all and BioWord2Vec use a vector size of 396 and 200, respectively, while our results show that it is possible to reach higher performances in concept and sentence similarity with a compressed vector of size 152. A reduced vector size should be preferred because it reduces the curse of dimensionality, improves generalizability, and requires fewer computational resources (more on the rationale behind every hyperparameter and their selected values is reported in Supplementary File S1, available as supplementary data at *Bioinformatics* online).

However, the CSpace vector size was determined with empirical experiments comparing different sizes, and further research is needed to uncover the relationship between performance and vector size.

**Figure 3.**
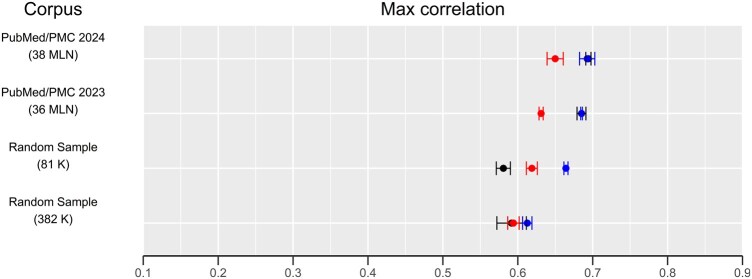
Forest plot of CSpace embedding model performances with growing corpus size, expressed as a number of articles between parentheses, on three test sets: MayoSRS (black), UMNSRS relatedness (red), and UMNSRS similarity (blue). Number of sentences in millions (M) and thousands (K). Performance is computed as the Pearson correlation between human and model similarity judgement (expressed as embedding vector cosine similarity). The horizontal bars represent the standard deviation among three repeated training/evaluation runs.

The limit of our approach is that it works well for single concepts and relatively short sentences, quickly degrading the performance as the length of the sentence increases. Popular transformer alternatives, on the other hand, excel at generating contextual embeddings for long sentences, paragraphs and even entire book chapters, as the OpenAI ada-v2 input limit is currently 8191 tokens, roughly equivalent to 15–20 pages of text in a novel. We argue that biomedical concept and sentence similarity do not necessarily need such complexity, and comparable or superior performance can be achieved with smaller, specialized models and carefully chosen algorithms, significantly reducing computational costs. Furthermore, we observe that the correlation between CSpace and human similarity judgments consistently improves with increasing training set size (Fig. 3), although a slight decrease is noted in the sentence similarity task (Table 7). This suggests room for further improvement as new biomedical literature and annotations become available.

While optimizing hyperparameters, we noticed that the hyperparameter that matters most is the number of training examples since models with equal training set size cluster together with few exceptions, regardless of the hyperparameters used. This behavior is clearly visible in Fig. 3, where we report the best CSpace performances among all tested hyperparameters on different datasets of increasing size. It is interesting to note, however, that the random sample of 81 000 papers had a slightly better average performance with respect to the random sample of 382 000 papers. On the other hand, the difference in performance between different test sets tends to decrease as the number of training examples increases. For this reason, we consider this a random effect due to the small training set size.

## Supplementary Material

btaf376_Supplementary_Data

## Data Availability

The code used for data pre-processing, training, visualization and evaluation of CSpace embeddings, all the experiments results and test benchmarks are available at https://doi.org/10.5281/zenodo.15125706 and in the GitHub repository https://github.com/cosbi-research/cspace; the PubMed data is available at https://www.nlm.nih.gov/databases/download/pubmed_medline.html, the PubMedCentral data is subdivided into manuscript collection available at ftp://ftp.ncbi.nlm.nih.gov/pub/pmc/manuscript, and open access commercial and non-commercial use (oa_comm and oa_noncomm) available at ftp://ftp.ncbi.nlm.nih.gov/pub/pmc, while the Pubtator3 data can be found in https://ftp.ncbi.nlm.nih.gov/pub/lu/PubTator3/ Bio and Med archives can be found on the S3 bucket https://www.biorxiv.org/tdm. The US clinical trials database is available at https://aact.ctti-clinicaltrials.org/snapshots directly as SQL data dumps. CSpace model in KeyedVectors (cspace.kv.bin) and compressed format (cspace.compressed.100k.bin) with training data up August 2024 is available at Zenodo (Fondazione The Microsoft Research - COSBI 2025). Alongside with CSpace, in the same location there is the co-occurrence model (cspace.bigrams.pkl) and concept-frequency model (cspace.dict.pkl). See the examples folder in the GitHub repository for a complete example on how to use them, and the preprocessing folder for the code to build the CSpace training data.
