# Supplementary material for: CSpace: a concept embedding space for biomedical applications
Source: Bioinformatics. 2025 Jun 27;41(7):btaf376. doi: 10.1093/bioinformatics/btaf376 (PMC12275461; doi:10.1093/bioinformatics/btaf376)
Supplement: btaf376_Supplementary_Data [file btaf376_supplementary_data.zip › Tomasoni_et_al_cspace_Supplementary_File_S1.pdf]

CSpace: A concept embedding space for bio-medical applications

Danilo Tomasoni, Luca Marchetti

Supplementary File S1

Table of Contents

Supplementary Methods 2

Hyperparameters meaning and discussion on final Chosen value 2

Performance measures 5

Vocabulary coverage 5

Concept and Sentence similarity 6

References 7

Supplementary Methods

In this section, we thoroughly explain the CSpace hyperparameters and the rationale behind the choice of each specific value, as well as the performance measures used in the main document.

Hyperparameters meaning and discussion on final Chosen value

CSpace was trained with the FastText algorithm, an improvement over the original Word2Vec algorithm, exploiting sub-word similarities to improve the embedding quality.

The FastText algorithm has many hyperparameters that affect the quality of the resulting embeddings. In general, we selected the hyperparameter values that maximize the chosen performance measures, that is, the correlation between the model and human expert similarity judgment (specifically Tables 5 and 7 of the main text).

However, below we discuss our intuition behind each hyperparameter function and the specific Chosen value. The actual GenSim hyperparameter name is reported between parentheses:

- **Number of epochs** (epochs): The training process consists of a configurable number of epochs. In each epoch, the ANN tries to predict the surrounding words of the target word for every sentence in the training set. Thus, with multiple epochs, the ANN will process each sentence a number of times equal to the number of epochs. It was noted that randomly shuffling the row order between epochs has a positive effect on the training process and the final embedding quality.
    - **Chosen value**: Experiments showed that a high number of epochs was of little help (if not harm) for learning good embeddings. The final choice of 5 was guided by other embedding models for different domains, where good results were achieved with just 3 epochs.
- **α** (alpha): The learning rate. This is the most influential hyperparameter and controls the ANN weights update speed. The higher the alpha, the faster the change in the embedding vector values. Setting a too-high alpha can cause instabilities in the training process, especially after the first few epochs.
    - **Chosen value**: The general recommendation is 0.025, while we set it to 0.03. The choice was purely driven by experiment results.
- **min α** (min_alpha): Between different epochs, the alpha hyperparameter decays linearly up to this value. It was noted that in earlier epochs, the ANN weights are random, and a higher alpha helps to get the ANN weights in the right neighbourhood. On the contrary, in later epochs smaller alpha helps the stochastic

gradient descent algorithm (the optimization algorithm used by FastText) to find the exact minimum of the cost function.

  - **Chosen value**: While we experimented with different min_alpha values, the default was retained as it was one that provided the best overall results.

- **Negative examples** (negative): FastText is a self-supervised learning method that tries to solve a classification problem constructed from the plain English sentences provided in input. Given a target word, the ANN should predict if another word appears in the target word's surroundings. The algorithm selects for each correct surrounding word, a number of wrong words, that are the negative examples, sampled randomly from the entire corpus.
  - **Chosen value**: While we experimented with different negative values (up to 40), the default was retained as it was the one that provided the best overall results. We observed that the performance of CSpace does not vary significantly with negative examples in the range of 5-10.
- **Skip-Gram** (sg): FastText can construct the classification problem with the skip-gram or the CBOW method. The skip-gram method is the one described for the Negative examples hyperparameter, while the CBOW tries to solve the opposite problem of predicting the target word from surrounding words. If this parameter is set to 1, the Skip-gram algorithm will be used; otherwise, CBOW.
  - **Chosen value**: While we experimented with CBOW mode, Skip-gram provided the best overall results.
- **Window size** (window): While constructing the classification problem, the algorithm should exclude surrounding words that are located at a distance from the target word, measured by the number of in-between words, greater than this specified value.
  - **Chosen value**: Our final choice (20) is significantly different from the default (5) and may look surprising since the average length of each sentence in the training dataset is about 21 words. One possible explanation is that our unique pre-processing steps may reframe the ANN task to focus on predicting biologically meaningful concept associations – such as in the sentence "Gene **Phex** encodes a … which suppresses … **Vitamin D** bioactivation". These kinds of associations are typically found farther apart within a sentence, which could justify the higher value. Another observation is that CSpace concept similarity judgment is not significantly affected by window size in the range of 5-20, while other authors (Zhang, Chen, Yang, Lin, & Lu, 2019) suggested that a high window size is more suited for intrinsic tasks, while a low window size is more suited for extrinsic tasks.

- **Vector size** (vector_size): The size of the vector that contains the embedding for a single concept.
    - **Chosen value**: As mentioned in the main text, one advantage of CSpace is that it uses significantly smaller embedding size (152) compared with typical alternatives (300 for Google news, 396 BioWord2Vec, 200 BERT-Crel-all, 1536 OpenAI ada-v2), featuring significantly smaller memory, storage and computational requirements. This was the main rationale behind this choice. Further, with fewer parameters, the chance that the embeddings are not actually learning but simply memorizing the training set is smaller. A technical reason is that the vector size should be a multiple of 4 for the highest efficiency in the underlying array operations.
- **Max length of char n-grams** (max_n): Max number of characters to include while computing sub-word similarities among different concepts.
    - **Chosen value**: while we experimented with different values, the default provided the best results overall.
- **Min length of char n-grams** (min_n): Min number of characters to include while computing sub-word similarities among different concepts.
    - **Chosen value**: while we experimented with different values, the default provided the best results overall.
- **Min word frequency** (min_count): Concepts with a total number of occurrences lower than this value are removed from the training set.
    - **Chosen value**: was kept the default (5). This is because, according to the experience of the GenSim creators, the down-sample threshold is much more effective at trimming common words.
- **Down-sample threshold** (sample): Not all words/concepts are treated equally in the training process. Very common concepts/words have little impact and discarding some of them improves at the same time the training speed and the embedding quality. For this reason, target words (w) are ignored in the training set with probability proportional to $1/\sqrt{p(w)}$, where p(w) is the target word w marginal probability of appearing in the training set.
    - **Chosen value**: Initially we experimented with stop-words removal through the popular SpaCy NLP package. However, we realized that performances benefit more from trying more sample values over performing more particular stopword removal. This is because sample thins whatever is overrepresented in the training set, rather than some external idea of which words are too frequent.
- **Number of workers** (workers): The number of CPU processes working in parallel to update ANN weights. More parallelism means more stochasticity in the results

since working on multiple different sentences from the training set at the same time led to a different ANN weights update for each process. Thus, it is possible that the contribution of a worker is cancelled out by the contribution of another one because the processes synchronize on the weights update step, which sum up all the process's contributions.

  - **Chosen value**: This hyperparameter was set to the maximum number of cores of the server where the training was running, to accelerate the training.

- **Phrases Threshold** (threshold): Controls the joining of two highly co-occurring words into a single bi-gram. The higher the threshold, the fewer words are joined together.
  - **Chosen value**: we experimented with different values and noticed that using different thresholds during training (1.0) and sentence similarity (5.0) improved the performances on the BIOSSES dataset.

Performance measures

In this section, we discuss the performance measures employed to validate CSpace in the tasks of vocabulary coverage, concept similarity and sentence similarity.

Vocabulary coverage

The number of concepts/words actually embedded in the embedding model is an often unrepresented measure of embedding quality.

However, we argue that it is important to measure not only the embedding quality, but also the number of concepts that can be actually embedded, because this is crucial to the real-world applicability of the model itself.

To this aim, in Table 1 we use the out-of-vocabulary ratio (OOV), that is the proportion between the number of concepts not embeddable with a given model and the total number of concepts in the test set itself. The higher the OOV, the higher the chance that the model won't encode a new concept of your choice.

Further, the test procedure computes the correlation between the model and human similarity judgment automatically excluding concept pairs that cannot be embedded by the model, thus potentially biasing the results toward models with very few high-quality embeddings.

Concept and Sentence similarity

The test procedure of Tables 5 and 7 of the main text aims at evaluating how much the model similarity judgment aligns with what is expected to be the judgment of a human expert in the field.

To do that we collected three datasets of concept pairs and one dataset of sentence pairs with a numerical measure of their similarity judged by a human expert.

Subsequently, we embedded each concept pair, and each sentence pair with the model and computed the cosine similarity between them.

The cosine similarity is a single floating-point number in the range [-1,1] that can be interpreted as

- -1 when the concepts are the inverse one of the other;
- 0 when the concepts are not related;
- 1 when the concepts are semantically equivalent.

Finally, we compute the correlation between this cosine similarity and the numerical measure judged by the human expert.

The higher the correlation, the more the model aligns with the human judgment.

References

Zhang, Y., Chen, Q., Yang, Z., Lin, H., & Lu, Z. (2019). BioWordVec, improving biomedical word embeddings with subword information and MeSH. *Scientific Data*. https://doi.org/10.1038/s41597-019-0055-0
