# Supplementary material for: CSpace: a concept embedding space for biomedical applications
Source: Bioinformatics. 2025 Jun 27;41(7):btaf376. doi: 10.1093/bioinformatics/btaf376 (PMC12275461; doi:10.1093/bioinformatics/btaf376)
Supplement: btaf376_Supplementary_Data [file btaf376_supplementary_data.zip › Tomasoni_et_al_cspace_Supplementary_File_S2.pdf]

CSpace: A concept embedding space for bio-medical applications

Danilo Tomasoni, Luca Marchetti

Supplementary File S2

	Idiopathic Pulmonary Fibrosis	Chronic Pulmonary Fibrosis	Chronic Obstructive Pulmonary Fibrosis	Chronic Interstitial Lung Disease	MESH: D054990
**Idiopathic Pulmonary Fibrosis**	1				
**Chronic Pulmonary Fibrosis**	0.95 (0.87)	1			
**Chronic Obstructive Pulmonary Fibrosis**	0.92 (0.83)	0.95 (0.92)	1		
**Chronic Interstitial Lung Disease**	0.91 (0.87)	0.91 (0.90)	0.89 (0.87)	1	
**MESH: D054990**	0.83 (0.68)	0.75 (0.65)	0.72 (0.60)	0.75 (0.63)	1

Supplementary Table S1. *Example of cosine similarity between concepts strictly related to "Idiopathic Pulmonary Fibrosis", including n-grams and MESH ID, of CSpace with training data up to August 2024 and February 2023 (shown between parentheses).*

MeSH ID	MeSH Term	CSpace Cosine Similarity Score
D011658	Pulmonary Fibrosis	0.932
D017563	Lung Diseases, Interstitial	0.921
D054988	Idiopathic Interstitial Pneumonias	0.916
D001989	Bronchiolitis Obliterans	0.874
D029424	Pulmonary Disease, Chronic Obstructive	0.870
D017565	Sarcoidosis, Pulmonary	0.873
D008171	Lung Diseases	0.871
C562470	Interstitial Pneumonitis, Desquamative, Familial	0.867
D000081029	Pulmonary Arterial Hypertension	0.864
D011656	Pulmonary Emphysema	0.863
D008173	Lung Diseases, Obstructive	0.863
D012595	Scleroderma, Systemic	0.847
D005355	HRTC - Fibrosis	0.859
D017564	Radiation Pneumonitis	0.857
D000080445	Asthma-Chronic Obstructive Pulmonary Disease Overlap Syndrome	0.857
D000542	Alveolitis, Extrinsic Allergic	0.856

Supplementary Table S2. *Most similar concepts to " Idiopathic Pulmonary Fibrosis" (MeSH: D054990) in vector space with CSpace, up to cosine similarity 0.85. All diseases reported share some clinical phenotype of the disease. CSpace was trained with data up to August 2024.*

	**Tuberculosis**	**Cavitary Tuberculosis**	**Mycobacterium**	**Tuberculin**	**MESH: D014376**
**Tuberculosis**	1				
**Cavitary Tuberculosis**	0.91 (0.89)	1			
**Mycobacterium**	0.84 (0.82)	0.73 (0.70)	1		
**Tuberculin**	0.79 (0.72)	0.73 (0.64)	0.70 (0.68)	1	
**MESH: D014376**	0.79 (0.77)	0.74 (0.67)	0.73 (0.71)	0.70 (0.67)	1

Supplementary Table S3. *Example of cosine similarity between concepts strictly related to “Tuberculosis”, including n-grams and MESH ID, of CSpace with training data up to August 2024 and February 2023 (shown between parentheses).*

ID	MeSH Term	CSpace Cosine Similarity Score
MESH:D014390	Tuberculosis, Meningeal	0.95
Taxonomy:182785	Mycobacterium tuberculosis	0.929
MESH:D014397	Tuberculosis, Pulmonary	0.929
MESH:D014379	Tuberculosis, Avian	0.916
MESH:D014381	Tuberculosis, Cardiovascular	0.914
MESH:D014391	Tuberculosis, Miliary	0.913
MESH:D014384	Tuberculosis, Female Genital	0.913
MESH:D014386	Tuberculosis, Hepatic	0.911
MESH:D014387	Tuberculosis, Laryngeal	0.904
MESH:D014380	Tuberculosis, Bovine	0.904
MESH:D014382	Tuberculosis, Cutaneous	0.903
MESH:D014383	Tuberculosis, Endocrine	0.903
MESH:D014385	Tuberculosis, Gastrointestinal	0.899
MESH:D009165	Mycobacterium Infections, Nontuberculous	0.895
MESH:D004654	Empyema, Tuberculous	0.894
MESH:D014388	Tuberculosis, Lymph Node	0.894
MESH:D014392	Tuberculosis, Ocular	0.892
MESH:D014396	Tuberculosis, Pleural	0.891
MESH:D014398	Tuberculosis, Renal	0.879
MESH:D014395	Peritonitis, Tuberculous	0.877

Supplementary Table S4. *Most similar concepts to "Tuberculosis" (MeSH: D014376) in vector space with CSpace, up to cosine similarity 0.85. All concepts reported share some clinical phenotype of the disease. CSpace was trained with data up to August 2024.*

Predicted association	Explanation	PMID
MAPT -> IPF	Our study found that the methylation levels of cg00045227 (OR8U8), cg00577578 (GBAP1), cg07163735 (**MAPT**), cg14222479 (ARPM1), and cg19263494 (PMF1) were causally associated with **IPF**.	39425105
MAPT->Long Covid	This study aims to demonstrate how **Long COVID** phenomena can be caused by the development of **tau protein**	36773054
AAVS1-> Lung Diseases -> IPF	Adeno-associated viral (**AAV)** vectors have been thought to be primary candidates for gene delivery in patients with **pulmonary diseases**	39581991
lipoprotein LpqT -> Tuberculosis	Wide genome screening of M. **tuberculosis** H37Rv mutants has allowed the identification of additional Lpps encoding genes specifically required for mycobacterial survival in vivo or in vitro. Several Lpps have been identified in both studies: **lpqT**, lpqY, lprG (described elsewhere), lpqZ and lprK.	23076359
CP -> Fibromyalgia	Thus, copper and **ceruloplasmin** may play a role in contributing to the oxidative stress pathogenesis of **fibromyalgia**.	36246401

Supplementary Table S5. *Gene-Disease example associations predicted by CSpace and confirmed a posteriori by literature search.*
